# Clinical Characteristics and Mortality-Associated Factors in COVID-19 Critical Patients in a Portuguese ICU

**DOI:** 10.7759/cureus.29610

**Published:** 2022-09-26

**Authors:** Laura Costa, José Martins, Marina Costa, Ana Isabel Oliveira, Dina Leal, Luís Lencastre

**Affiliations:** 1 Critical Care, Serviço de Medicina Intensiva, Hospital de Braga, Braga, PRT; 2 Internal Medicine, Hospital of Braga, Braga, PRT; 3 Critical Care, Hospital de Braga, Braga, PRT; 4 Intensive Care Medicine, Hospital of Braga, Braga, PRT; 5 Critical Care, Hospital of Braga, Braga, PRT

**Keywords:** ventilation, comorbidity, critical illness, sars-cov-2, covid-19

## Abstract

Introduction: Severe COVID-19 is associated with serious complications and poor outcomes. Older age and underlying comorbidities are known risk factors for severe COVID-19, but a better understanding of baseline characteristics and outcomes of patients with severe COVID-19 is urgently needed.

Methods: This study was a retrospective case series of 227 consecutive patients with laboratory-confirmed COVID-19 admitted to the intensive care unit (ICU) at our institution between March 2020 and December 2021. Demographic and clinical data were collected.

Results: The median age of patients was 65 years, and 180 (79.3%) were male. Cardiovascular comorbidities were frequent and included hypertension (n=148; 65.2%), dyslipidemia (n=116; 51.1%), obesity (n=114; 50.2%), and diabetes mellitus (n=80; 35.2%). About 20% of the patients had the chronic respiratory disease, with sleep apnea being the most common. Immunosuppression was identified in 13% of the patients, with autoimmunity, post-transplantation, and neoplasms being the most represented causes. Most patients were admitted to the ICU at six to 15 days after symptom onset, corresponding to stages IIb (pulmonary involvement/hypoxia) and III (hyperinflammatory). All patients received systemic steroids, with an average treatment duration of 22 days. Several ventilatory support strategies were used; 80 patients were supported entirely noninvasively with high flow nasal oxygenation and noninvasive ventilation, while 164 patients were invasively ventilated. Most intubations (65%) occurred in the first 24 hours after admission, and the mean duration of mechanical ventilation was 14 days. The reintubation rate was 10%, occurring on average two to three days after planned extubation. Thirty-two tracheostomies were performed. Bacterial co-infection was treated in 75% of patients, and *Aspergillus* co-infection complicating COVID-19 pneumonia was diagnosed in eight patients. Median ICU and hospital stays were 15 and 25 days, respectively, and the 28-day mortality rate was 38%. Patients over 75 years experienced a higher mortality rate (56%). Increased age and multimorbidity, particularly comprising cardiovascular disease and associated risk factors, were significantly more common in patients who died within 28 days after ICU admission.

Conclusions: A large proportion of critically ill COVID-19 patients required prolonged mechanical ventilation. ICU/hospital stay and mortality were particularly elevated in older patients and patients with cardiovascular risk factors. Considerable discrepancy existed between the proportion of patients with microbiological documentation of bacterial infections and those receiving antimicrobials. Improved methods for adequate microbiological diagnosis are needed and stewardship programs should be reinforced.

## Introduction

The global COVID-19 pandemic continues to affect international healthcare. To date, the World Health Organization has recorded more than 460 million cases and more than 6 million deaths have been confirmed worldwide [1]. In Portugal, there have been over three million confirmed infections and more than 21,000 deaths as of April 2022 [2]. COVID-19 is mainly characterized by respiratory symptoms of varying severity, ranging from asymptomatic or mildly symptomatic to severe respiratory failure or acute respiratory distress syndrome (ARDS). In severe cases, multi-organ failure may occur [3,4].

Multiple risk factors, such as advanced age and male gender, have been proposed as being associated with a higher risk of developing severe COVID-19 [5]. Additionally, conventional cardiovascular risk factors, including hypertension, obesity, and diabetes, along with established cardiovascular disease appear to be associated with a more severe course of the disease and higher mortality [4,6].

Intensive care units (ICUs) have an important role in managing patients in critical conditions. ﻿The proportion of patients requiring admission to an ICU has ranged between 4% and 32% [7,8]. Available data from systematic reviews and meta-analysis point to high mortality rates among ICU patients, varying between 35% and 42% [9]. An urgent need exists to better understand baseline patient characteristics and outcomes of severe COVID-19. In the current study, we aimed to characterize COVID-19 patients admitted to the ICU at our institution.

## Materials and methods

We conducted a retrospective case series of all consecutive patients with severe forms of COVID-19 admitted to the ICU of Hospital de Braga from March 2020 to December 2021, and we included follow-up outcomes, such as mortality and time spent in the hospital, until March 2022.

In the pandemic setting, Braga’s hospital was designated as the COVID-19 reference center for the Minho region, and, as such, critically ill COVID-19 patients within the region were transferred there. ICU admission occurred at the discretion of the attending critical care physician, but general criteria included patients requiring rapid increase oxygen supplementation, noninvasive positive pressure ventilation, invasive mechanical ventilation, or vasopressors.

Data were obtained retrospectively from electronic medical records. The collected information included demographic data, laboratory and imaging data, information on organ dysfunction and support, SAPS II and APACHE II scores, pharmacological strategies, and clinical outcomes. Categorical variables are presented as frequencies and percentages, and continuous variables as means and standard deviations, or medians and interquartile ranges for variables with skewed distributions. Normal distribution was checked using the Shapiro-Wilk test or skewness and kurtosis. We compared continuous variables between groups using a t-test for independent samples (for normal distributions) and a Mann-Whitney U test (for skewed distributions), and we used the chi-square test to compare categorical variables.

## Results

This case series included 227 critically ill patients with SARS-CoV-2 infection and severe respiratory involvement. The data from the first and second epidemic waves are analyzed together in view of the fact that a relatively constant flow of patients was maintained from March to December 2020 n in our ICU, without any significant affluence differences having been noted. a total of 124 patients were admitted during the first and second waves, and 103 patients were admitted over the third epidemic wave (from late December 2020 to March 2021).

**Figure 1:**
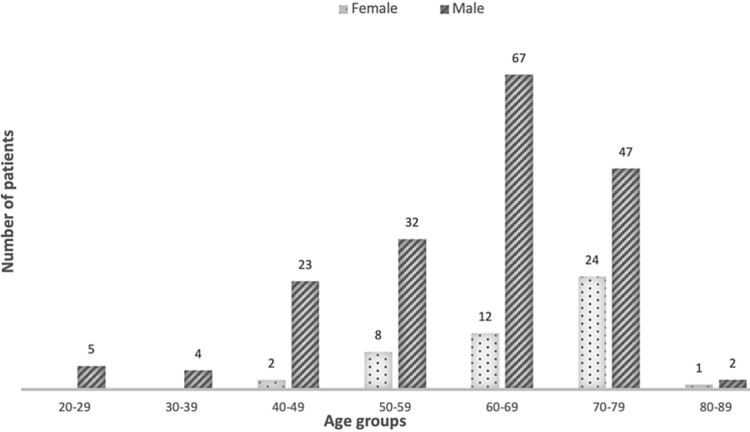
Distribution of patients according to gender and age group

Baseline demographic and clinical characteristics are shown in Table 1. The average age of the patients was 62.9 (SD 11.7) years, ranging between 24 and 83 years. Overall, 47 (20.7%) patients were female. The male gender was more represented across all age groups, as shown in Figure 1. The median age was slightly higher among women as compared with men (69 vs 63 years). There were no significant differences in age and gender distribution between epidemic waves.

**Table 1:** Baseline demographic and clinical characteristics Data are presented in n (%), mean (SD) and median (IQR). IQR, interquartile range; ICU, intensive care unit; SD, standard deviation

Descriptor	N (%) of patients
﻿Demographic description	
Age, mean (± SD)	62.9 (± 11.7 ) years
Male gender (%)	180 (79.3%)
Comorbidities and Lifestyle Behaviors	
Hypertension, n (%)	148 (65.2)
Dyslipidemia, n (%)	116 (51.1)
Obesity, n (%)	114 (50.2)
Diabetes Mellitus, n (%)	80 (35.2)
Chronic Respiratory Disease, n (%)	41 (18.1)
﻿Cardiovascular Disease, n (%)	30 (13.2)
Chronic Kidney Disease, n (%)	14 (6.2)
﻿Current Tobacco Smoker, n (%)	14 (6.2)
Active Malignancy, n (%)	10 (4.4)
Chronic Alcohol Abuse, n (%)	9 (4.0)
Heart Failure, n (%)	9 (4.0)
Time from symptom onset to ICU admission, median (IQR)	6 (2-10.5)
APACHE II score at admission (PMR%)	40.5 (39.7)
SAPS II score at admission (PMR%)	17.5 (26.5)
﻿Outcome	
﻿ICU length of stay, median (IQR)	25 (5-47.5) days
Hospital length of stay, median (IQR)	15 (6-26) days
Mortality at ICU discharge, n (%)	84 (37.0)
Mortality 28 days after ICU admission, n (%)	86 (37.9)
﻿Readmission to ICU within 48 hours, n (%)	3 (1.3)

A total of 203 (89.4%) patients had at least one medical comorbidity. The most common chronic medical problems were related to cardiovascular risk factors, including hypertension (148 patients, 65.2%), dyslipidemia (116 patients, 51.1%), obesity, defined as BMI above 30 kg/m^2^, (114 patients, 50.2%), and diabetes mellitus (80 patients, 35.2%). About 18% of the patients had chronic respiratory disease, with obstructive sleep apnea being the most common (19 patients), followed by asthma (13 patients).

A chronic condition causing immunosuppression was identified in 29 patients (13%) in this case series. The most frequent underlying conditions were autoimmunity (16 patients), active malignancy (10 patients), and post-transplantation (three patients). Rheumatoid arthritis was the most common autoimmune disease, and three patients had recently been diagnosed with active hematological malignancies. The relative frequency of immunosuppression related conditions was higher in the third epidemic wave (20 patients) as compared to the previous period (nine patients). 

**Figure 2:**
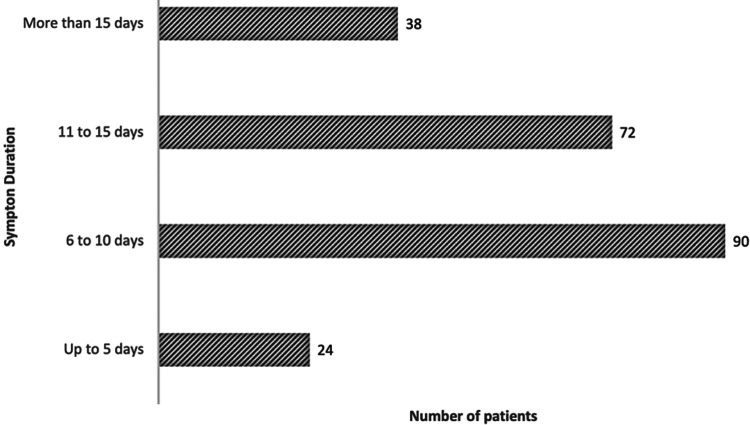
Distribution of patients according to COVID-19 symptom duration

Time of disease progression, counted from symptom onset, is a prominent criterion in clinical management. The median duration of symptoms before ICU admission was six days (IQR 8.5). Figure 2 shows the distribution of patients by duration of symptoms attributable to COVID-19 at ICU admission. Most patients were admitted six to 15 days after symptom onset, corresponding to stages IIb (pulmonary involvement/hypoxia) and III (hyperinflammatory). Fatalities at 28 days post ICU admission were more frequent in patients with longer times of disease progression. Those with symptoms for more than 15 days prior to ICU admission had a mortality of 70.8% (17 deaths), and those with symptom duration ranging from six to 10 days had a mortality rate at 28 days of 54.2% (39 deaths). The 28-day mortality rate of patients with symptoms lasting up to five days had a mortality of 18.4% (seven deaths).

COVID-19 therapeutics varied significantly as the pandemic evolved and scientific evidence increased. At the beginning of the pandemic, hydroxychloroquine was used in 28 patients, but it was not used at all after that in accord with data from clinical trials that showed it was ineffective for treating COVID-19. Azithromycin was also used at an early stage in 25 patients. Remdesivir was similarly used throughout the first/second and third epidemic waves, having been administered to 40 patients in total (17.6%), and at the time of writing, it remains a valid therapeutic option in early stages of infection. In our case series, remdesivir treated patients had a 28-day mortality of 35%, slightly under global mortality rate of 37.9%. Lopinavir-ritonavir, a type 1 inhibitor of the HIV aspartate protease, was used in two patients in this case series. Tocilizumab was used in four patients admitted during the last few months of 2021, when the drug became available in our center. Steroids are used to reduce the upregulated inflammatory response typical of SARS-CoV-2 infection. Systemic steroids were used in 102 patients in the first and second epidemics waves, corresponding to approximately 82.2% of patients admitted within that time frame. All patients received systemic steroids from late December to March 2021. On average, steroid therapy beginning was on day 8 of symptomatic disease for a duration of 22 days.

**Figure 3:**
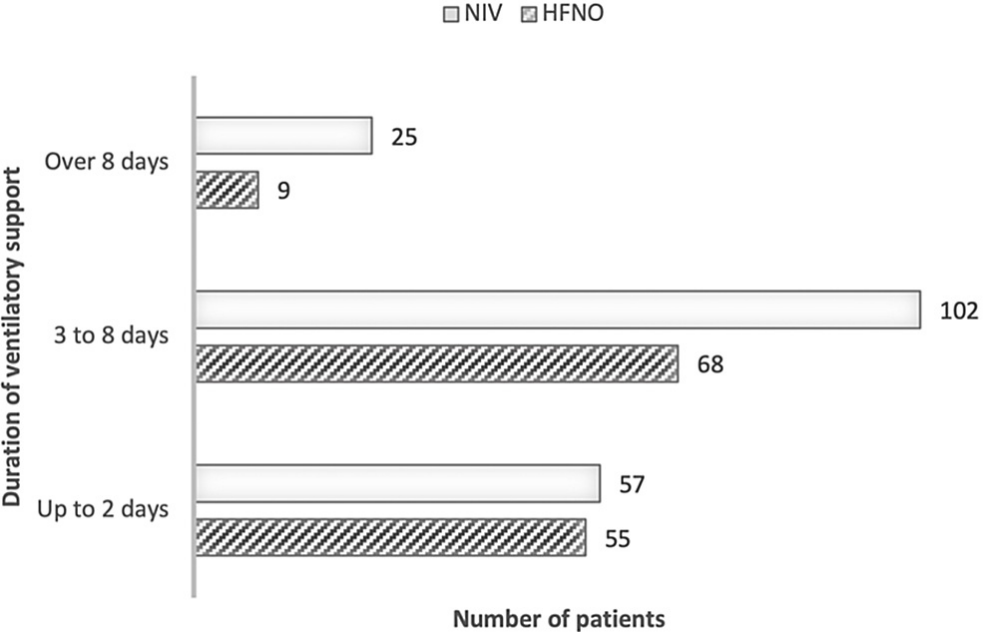
Distribution of patients according to the duration of non-invasive ventilatory support

Several ventilatory support modalities were used. Most patients were managed noninvasively for at least part of their ICU stay, the majority before progression of hypoxemia and intubation for invasive mechanical ventilation. Exclusive noninvasive ventilatory support, either high-flow nasal oxygenation (HFNO), noninvasive ventilation (NIV), or both, was used in 65 patients (28.6%). Ventilatory support management changed with the evolution of the pandemic with a larger proportion of patients being managed entirely noninvasively over the third wave (38.9%, n= 40) as opposed to 20.2% (n= 25) during the first and second epidemic waves. The 28-day mortality rate in patients whose hypoxemia was managed entirely noninvasively was 12.3% (eight patients). Death occurred in half of the patients that underwent invasive mechanical ventilation (81 patients, 50%), regardless of whether they had received non-invasive ventilation prior to intubation. Figure 3 illustrates the distribution of patients according to the duration of noninvasive ventilatory support, either with noninvasive ventilation (mean seven days) or high-flow nasal oxygenation (mean three days).

Invasive mechanical ventilation occurred in 164 patients (72.2%). Most intubations (65%) occurred in the first 24 hours after ICU admission, but several patients were intubated relatively later in their ICU stay, which explains why the average initiation of invasive mechanical ventilation occurred approximately on day 4 after admission. In 31 patients, invasive mechanical ventilation was initiated later, after day 5 in the ICU. Mean duration of mechanical ventilation was 14 days, ranging from one to 67 days. The proportion of time under invasive mechanical ventilation was on average 52.8% of the total length of stay. The reintubation rate was 10% (22 patients), occurring on average two to three days after planned extubation. Patients with associated ARDS often remained under invasive ventilation for difficult and prolonged weaning, thus requiring tracheostomy. A total of 32 tracheostomies were performed. Relatively more tracheostomies were performed during the third epidemic wave (18 as opposed to 14 during the first and second waves). Most of the procedures took place in the operating room owing to concerns related to aerosol generation during the first and second waves (85.7%, n= 12). In contrast, over the third epidemic wave, most were percutaneous tracheostomies with minor procedure adjustments to minimize the spread of aerosols. The procedure occurred on average at day 26, ranging between day 12 and day 44.

Bacterial respiratory co-infection was assumed in 173 patients (76.2%), based on clinical and laboratory criteria. Diagnosis of co-infection and initiation of antimicrobials occurred, on average, 12 days after the initial viral infection symptoms appeared. In most patients (n=117, 67.6%) no etiologic agent was identified on respiratory samples, either by culture or molecular methods. In cases in which microbiological identification was achieved, gram-negative bacteria prevailed, particularly from the Enterobacterales order: Klebsiella pneumoniae, Pseudomonas aeruginosa, and Haemophilus influenzae were the most frequently isolated, in descending order. Twenty-two gram-positive organisms were identified (primarily Staphylococcus aureus, Streptococcus pneumoniae, and Enterococcus faecalis), corresponding to approximately one-third of positive cultural samples (endotracheal aspirates). Fungal co-infection, particularly by Aspergillus, has been described as a complication of severe and prolonged COVID-19 pneumonia and was treated in eight patients (Table 2).

**Table 2:** Management in the ICU and clinical outcomes Data are presented in n (%)

Variable	N. (%) of patients
﻿ICU therapy	
Noninvasive positive-pressure ventilation	80 (35.2%)
﻿Invasive Mechanical ventilation	164 (72.2%)
Extracorporeal membrane oxygenation	3 (1.3%)
Continuous renal replacement therapy	2 (0.9%)
﻿Pharmacologic therapy	
﻿Steroids	227 (100%)
Hydroxychloroquine	28 (12.3%)
Azytromicin	25 (11.0%)
Lopinavir/Ritonavir	2 (0.9%)
Remdesivir	40 (17.6%)
Tocilizumab	4 (1.8%)
Antimicrobial therapy	191 (84.1%)
Anticoagulation	98 (43.2%)

A total of 191 patients (84.1%) admitted for COVID-19 were treated with antibiotics during their ICU stay. The mean duration of antimicrobial therapy was 14.5 days, corresponding to a proportion of 66.8% antibiotic-days per days of ICU stay. De-escalation, based on microbiological results, occurred in 42 patients (35.3%). In addition to respiratory infections, other nosocomial infections were recorded in 37 patients, mostly catheter-associated urinary tract infections, identified in 29 patients (12.8%).

Disorders of coagulation and fibrinolysis were numerous and severe, and they were more common during the second COVID-19 outbreak. Full dose low-molecular-weight heparin (LMWH) was administered in just under half of patients (n=98; 43.2%). In 60 patients, LMWH was used owing to severe and disproportionate hypoxemia that raised concern for possible contribution of undiagnosed thromboembolic disease or microthrombi in the pulmonary circulation. Full-dose LMWH was initiated in 10 patients after formal imagiological diagnosis of pulmonary thrombus/embolus and in 24 patients because of arrhythmia with formal indication, mostly atrial fibrillation and flutter, with the majority of cases diagnosed in the ICU. Despite the preventive strategies, adverse outcomes were recorded in several patients. Five patients died from obstructive shock, which was assumed to be secondary to massive pulmonary embolism refractory to fibrinolysis; three patients had a thrombus in the abdominal aorta and ensuing critical limb ischemia, with vascular intervention and limb amputation being performed in two cases; six patients required endovascular/surgical recanalization and/or systemic anticoagulation for arterial occlusion and limb ischemia; and one patient died as a result of mesenteric ischemia. In some cases, several thrombotic phenomena occurred simultaneously, sometimes involving both venous and arterial circulation. The proportion of COVID-19 patients receiving anticoagulation was higher during the third epidemic wave (50.5%, n= 52) as compared to the first/second waves (37.1%, n= 46).

The median ICU and hospital stay was 15 days and 25 days, respectively. Global mortality at discharge and 28 days after ICU admission was 37.0% and 37.9%. Minor differences in mortality and length of stay are noticeable between the first/second and third epidemic waves. Mortality both at discharge and 28 days after ICU admission was 37.0% (n= 46) and 38.7% (n=48) on the first/second waves, slightly higher than those observed during the third epidemic wave: 34.9% (n=36) and 35.9% (n=37), respectively. Patients remained in the ICU for approximately three more days and a further five days in the hospital during the third wave, as compared to the previous period.

We compared age and sex differences between COVID-19 patients alive within 28 days of ICU admission and patients who died. Deceased patients were older (68.03 versus 59.8 mean age, p < 0.00001), and men were more represented (83.7% versus 72.1%, p=0.036). Deceased patients were more likely to have two or more comorbidities compared with patients alive within 28 days post ICU admission (91.8% vs. 79.4%; p = 0.04). A higher proportion of deceased patients had baseline cardiovascular risk factors, such as hypertension (74.5% vs. 50.0%; p = 0.0002), dyslipidemia (60.5% vs. 45.4%; p = 0.028), or diabetes mellitus (55.8% vs. 22.7%; p < 0.00001). Moreover, chronic respiratory comorbidities and established cardiovascular disease were significantly more common in the group in which death occurred within 28 days. There were no significant differences in proportion of obesity or immunosuppression between both groups (Table 3).

**Table 3:** Baseline demographic and clinical characteristics between the discharged and deceased patients Data are presented in n (%), mean (SD) and median (IQR). QR, interquartile range; ICU, intensive care unit; SD, standard deviation

﻿	All patients (n = 227)	Alive within 28 days of ICU admission (n = 141)	Dead within 28 days of ICU admission (n = 86)	P-value
Age, mean (± SD)	62.9 (± 11.7)	59.8 (± 12.3)	68.03 (± 8.6)	< 0.00001
Gender, n (%) Male Female	180 (79.3) 47 (20.7)	118 (83.7) 23 (16.3)	62 (72.1) 24 (27.9)	0.036485
Number of comorbidities, n (%)				0.044572
None	14 (6.2)	11(7.8)	3 (3.5)	
One comorbidity	22 (9.7)	18 (12.8)	4 (4.7)	
Two or more comorbidities	191 (84.1)	112 (79.4)	79 (91.8)	
Hypertension, n (%)	148 (65.2)	105 (74.5)	43 (50.0)	0.000174
Dyslipidemia, n (%)	116 (51.1)	64 (45.4)	52 (60.5)	0.027514
Obesity, n (%)	114 (50.2)	76 (53.9)	38 (44.2)	0.155589
Diabetes Mellitus, n (%)	80 (35.2%)	32 (22.7)	48 (55.8)	< 0.00001
Chronic Respiratory Disease, n (%)	41 (18.1%)	35 (24.8%)	6 (7.0%)	0.000698
﻿Cardiovascular Disease, n (%)	30 (13.2%)	1 (0.7%)	29 (33.7%)	< 0.00001
Imunossupression, n (%)	29 (12.8%)	23 (16.3%)	6 (7.0%)	0.065914
Invasive Mechanical Ventilation, n (%)	164 (72.2%)	86 (61.0%)	78 (90.7%)	0.005422
Antimicrobial therapy, n (%)	191 (84.1%)	109 (77.3%)	82 (95.3%)	0.000083
ICU length of stay, median (IQR)	25 (5-47.5) days	12.5 (6-27.5)	16 (5.25-25.5)	0.75656
Hospital length of stay, median (IQR)	15 (6-26) days	30 (17-67)	20 (8.5-32.5)	< 0.00001

Five of the patients admitted for SARS-CoV-2 infection had a full dose vaccine regimen (two shots at that time). In this subgroup only one patient received invasive mechanical ventilation, having died within 44 days of hospital stay - this was a case of previously diagnosed brain lymphoma, who was immunocompromised due to recent chemotherapy treatment. There were no other reported deaths in this group and the medium length of hospital and ICU stay was 28 and 16 days, respectively.

Another six patients were infected and developed severe disease after only one administration of anti-SARS-CoV-2 vaccine. Five of them were intubated for invasive ventilatory support for 8.7 days, on average, and death occurred in two patients.

Variant characterization was possible in 31 patients. Eight patients presented between January and June 2021 with the Alpha variant, and 23 presented with the Delta variant, admitted from July to December 2021. Those infected with the Alpha variant were on average 61.6 years old. The medium hospital and ICU stay was 17 and seven days, respectively, and two patients died in the ICU, one of which was previously immunocompromised due to recent chemotherapy. Three patients were intubated for mechanical ventilation and one needed ECMO support. Patients in which the Delta variant was identified were 59.7 years old, on average, relatively younger than the general COVID population. Medium hospital and ICU stay was 20 and eight days, respectively. Approximately half of Delta patients needed invasive ventilation (43.5%, n=10), and mortality rate within this subgroup was 34.8% (n=8) and even higher in the non-vaccinated population (37.5%, n=6).

## Discussion

We have presented the main demographic and clinical features of 227 patients with severe COVID-19 that were managed in our ICU. The demographic descriptors were in line with the literature evidence. Older individuals and males appear to be more prone to severe COVID-19 manifestations and ICU admission [3,10]. In addition, patients in our study were similar in age, degree of comorbidities, and reported severity of the illness to those reported in the literature [3,11,12]. A common denominator of patients with severe SARS-CoV-2 infection in this study was the co-existence of multiple conditions, especially cardiovascular diseases, including hypertension, type 2 diabetes, and ischemic heart disease [4,6,12].

Age is prominent in the literature as a frequently reported independent factor associated with both ICU admission and in-hospital mortality, along with male sex [13,14]. As the pandemic progressed, immunosuppression emerged as a frequent feature of patients at higher risk for severe disease [14]. In this case series, both ICU and 28-day mortality increased markedly with age. Patients over 75 years old had a 28-day mortality rate of approximately 56% as opposed to 17% for patients younger than 55

The therapeutic options targeting COVID-19 have varied significantly in parallel with the evolution of the pandemic, the availability of scientific evidence, and the variation in infection characteristics over time [9,15]. Accumulating evidence indicates that therapeutic choices depend on the disease phase. Therefore, remdesivir and other antivirals targeting active replication may have a significant role in the early stage of SARS-CoV-2-associated pneumonia, through reduction of the viral load, which is postulated to prevent a systemic inflammatory reaction and, in particular, alveolar damage. In contrast, the late pulmonary and hyperinflammatory phases are dominated by immunological processes. In this latter stage of infection, antiviral therapy strategies are unlikely to be effective, whereas anti-inflammatory drugs may be beneficial [7,16].

At the beginning of the pandemic, hydroxychloroquine and azithromycin were used, the latter for their immunomodulatory and anti-inflammatory effect. As no modification of mortality or clinical severity of disease could be demonstrated, the use of either drug in COVID-19 patients was discontinued. Subsequently, remdesivir became available in some countries, including Portugal, as a therapeutic approach for infection in an early stage of the disease. Remdesivir inhibits the synthesis of viral RNA by blocking RNA polymerase through competition with its natural analog, adenosine triphosphate, leading to delayed chain termination and thus inhibition of viral replication [17]. Remdesivir may decrease the risk of clinical worsening in terms of the need for invasive mechanical ventilation and may also decrease the rate of serious adverse events at up to 28 days [16,18]. In our case series, the 28-day mortality rate of remdesivir-treated patients was 35%, which was slightly lower than the global mortality rate of 39.1%.

As the pandemic progressed, attention focused on lopinavir-ritonavir, a type 1 inhibitor of the HIV aspartate protease. An in vitro inhibitory effect on SARS-CoV-2 was reported, with clinical and radiological improvement in some animal models. In our case series, lopinavir-ritonavir was only used in two patients, who were discharged home on a 28-day follow-up. Accumulating scientific evidence failed to demonstrate any significant benefit in reducing hospitalization, mortality, or functional outcomes in COVID-19, and lopinavir-ritonavir was no longer used.

In general, patients with ARDS requiring mechanical ventilation and ICU admission progress to the inflammatory phase of infection, and anti-inflammatory drugs may be beneficial in such patients. Therefore, most patients in our case series were prescribed remdesivir during ward stay, prior to admission to the ICU. However, inflammation inhibitors were used during the ICU stay. These included steroids, which were used in all patients, and tocilizumab, which was administered in four patients. Interleukin (IL)-6 antagonists have been used on the assumption that their immunosuppressive properties could control immune dysfunction and inflammation and reduce the duration and/or severity of COVID-19.

Tocilizumab is a recombinant monoclonal antibody against the IL­-6 receptor, which has been used to reduce cytokine release in several rheumatological pathologies. It is recommended for progressive severe or critical COVID-19 patients on the assumption that their immunosuppressive properties, and capability to mitigate the cytokine storm syndrome associated with severe COVID­19 pneumonia, could control immune dysfunction and inflammation and reduce disease duration and/or severity. Some evidence from systematic reviews and clinical trials suggests that its use in severe COVID-19 may be associated with a lower risk of mortality or mechanical ventilation [15,19]. Four patients in our study received tocilizumab, with administration occurring early in the ICU course, typically on the day of admission for ICU support, and a median of 10 days since the beginning of self-reported symptoms. Although this sample is too small to allow valid conclusions to be drawn, it is worth noting that all four patients were alive and dismissed from the hospital at a 28-day follow-up. Sarilumab, another IL-6 receptor antagonist, was not available for use in our center.

A total of 164 patients (72.2%) were intubated for invasive mechanical ventilation during their ICU stay. The proportion of invasive mechanically ventilated COVID-19 patients described in the literature varies between 40% and 90%, and our results are in line with most of the published reports [3,11,20,21]. A significant proportion of patients (n=31, 18.9%) were intubated relatively later, after 5 days in the ICU. These patients were mainly those with immunosuppression. Because they were at high risk of infectious complications associated with intubation and invasive ventilation, intubation was avoided to the greatest extent possible.

Although this case series is a descriptive study in a limited sample and it cannot provide definitive conclusions, there was a striking difference in 28-day mortality between patients whose respiratory failure was managed solely noninvasively (12.3%) and those who were subjected to invasive mechanical ventilation (50%). This difference may be explained, not only by the higher severity of the underlying disease of patients that needed invasive mechanical ventilation but also by ventilator-associated complications, which are a known cause of morbidity and mortality. The mortality observed in our invasively ventilated patients group was similar to that described in a recent meta-analysis [22]. Ventilatory support management changed with time with a larger proportion of patients being managed entirely noninvasively during the third wave. This is partly explained by the acquisition of HFNO by our ICU during the latter part of 2020. 

Tracheostomy was often used to manage patients that required prolonged invasive mechanical ventilation, as well as to address difficult weaning and facilitate rehabilitation and functional recovery. Conflicting recommendations exist about case selection, timing, and technique. Although large studies and meta-analyses in recent years have indicated that early tracheostomy may be associated with better outcomes, including more ventilator-free days, shorter ICU stays, less sedation, and reduced long-term mortality compared with late tracheostomy (after 10 days of intubation), recommendations from consensus working groups and expert opinion suggest a different direction for ventilated COVID-19 patients [23]. The suggested window for tracheostomy is between ICU days 10-21, and the recommendation is to delay the procedure until at least day 10 of mechanical ventilation and only when patients are showing signs of clinical improvement [24]. In our experience, tracheostomies were performed on average, on day 26 after intubation. The delay relative to our general ICU population (13 days post-intubation) and the recommendations may be explained by respiratory failure and the need for aggressive mechanical ventilation and prolonged pruning, as well as frequent use of drugs affecting coagulation and platelet function, implying more surgical tracheostomies, which are more subject to delays.

Although acute kidney injury has been described as an important complication of COVID-19, with a multicenter cohort study showing that nearly 20% of acute kidney injury patients required renal replacement therapy, in our sample only three patients needed such treatment for acute kidney injury [25-28].

Data on associated infections in COVID-19 patients are scarce, and the available studies are inconsistent, with relatively small cohorts. Current evidence points to the widespread use of antimicrobials in COVID-19 as a common practice, with frequent use of broad-spectrum agents [29]. However, most studies, mainly retrospective and single-centered, have highlighted that the incidence of bacterial respiratory co-infections at admission is low, affecting about 3.5% of critical patients, while the majority of infections are nosocomial, generally developing 10-15 days after ICU admission [30,31]. Higher rates of bacterial infections are reported in the most severe cases of COVID-19, with prolonged ICU stays. Therefore, although co-infections by community bacteria such as Listeria pneumophila or pneumococcus are rare, severely ill patients, particularly those staying in the ICU for long periods, seem to develop bacterial/fungal co-infections more frequently (in some series 40%-50%), particularly Enterobacterales and Aspergillus fumigatus, compared with patients with less severe forms of the disease [32,33]. In our series, gram-negative bacteria were the most frequently isolated agents. Gram-negative predominance, particularly of the Enterobacterales order, including K. pneumoniae, P. aeruginosa, and H. influenzae, as agents of respiratory coinfection in COVID-19 patients, is in line with the epidemiology of hospital and ICU infections in the past few decades [34,35]. In our case series, antimicrobial therapy was significantly more common in the group in which death occurred, as opposed to patients that were alive 28 days post ICU admission (95.3% vs. 75.3%; p = 0.00008). This may reflect increased mortality from nosocomial infections, or simply be a marker of more severe ARDS, which was often the reason for receiving antimicrobials, even if it was only for limited periods until no microbiological infection was documented.

The COVID-19 population was under great antimicrobial pressure, because of prolonged ICU hospitalization and the extensive use of broad-spectrum antimicrobial drugs [31]. One reason for this situation was the inability to isolate and identify microbial pathogens. We observed that timely microbiologic reports allowed appropriate antibiotic adjustment to a narrower spectrum in only 42 patients.

Additional nosocomial infections assumed great importance in COVID-19 patients, given the prolonged hospital stay and extensive use of antimicrobials and invasive devices and techniques, such as invasive mechanical ventilation, central lines, or urinary catheters. Nosocomial nonrespiratory infections were recorded in 37 patients, mostly catheter-associated urinary tract infections, which were diagnosed in 29 patients (12.8%). Severe nosocomial infections are associated with a poor prognosis for patients in ICUs, and our data seemed to confirm this association [36]. Among the patients with nonrespiratory nosocomial infections, 16 (55.2%) died within 28 days post ICU admission and the median ICU and hospital stay was 36.5 and 48 days, respectively.

Increased incidence of antimicrobial resistance has been globally reported, particularly in sites with a high burden of severe or critical COVID-19 [37-41]. Multiple causative factors have been proposed, and these are not only related to the use of broad-spectrum antimicrobials and consequent negative ecological impact but also the high intensity of care, use of prone position with extended and prolonged contact with a patient by multiple professionals, and the increase of healthcare personnel without experience in an ICU setting regarding contact precautions [37].

Increasing age and multimorbidity, particularly cardiovascular disease and associated risk factors, have been highlighted as independent risk factors for ICU admission and mortality in COVID-19 patients [42-44]. Despite having all the typical limitations of any retrospective study, our results illustrate the effect of baseline morbidity factors such as increasing age and chronic pathology, particularly diabetes, hypertension, and cardiovascular disease, on the mortality of patients with severe COVID-19. However, we did not find significant differences in obesity percentages between surviving and deceased patients within 28 days after ICU admission. Although obesity has been highlighted as a risk factor for ICU admission, it does not seem to be a factor for ICU mortality [43]. In fact, moderate obesity may have a protective effect in COVID-19 patients admitted to the ICU, with a lower risk of death having been reported in this patient population [45].

The demographic characteristics and burden of comorbidities of COVID-19 patients during the third epidemic wave were similar in our study. The treatments they received during the third pandemic wave differed from those of the first two waves in view of the higher use of noninvasive ventilation strategies, systemic steroids, and anticoagulation. This is in line with most data from other ICUs [46,47]. However, ICU and in-hospital mortality were lowest during the third wave, at the expense of longer lengths of stay. Studies comparing the prognosis of critical patients requiring mechanical ventilation throughout different waves documented that, even though the proportion of patients requiring invasive ventilation was smaller as compared to first and second waves, ICU and in-hospital mortality remained unimproved or increased in critical COVID-19 patients in the third epidemic wave [46,47]. Possible explanations for these results could be the higher number of ICU beds made available and, therefore, the admission of less severe patients; and the higher use of NIV and HFNO (the latter only became available at our center by the end of 2020) which resulted in fewer patients been intubated. This, however, would have to be the subject of a thorough analysis and possibly, another study.

In late 2020, the emergence of variants that posed an increased risk to global health prompted its characterization to prioritize pandemic monitoring and research. Until the time of writing, WHO has classified five "variants of concern" (VOC). A variant is considered to be of concern when one or more of the following conditions occur: increased virus transmissibility, increased virulence or clinical change in the disease, and decreased effectiveness of social and public health measures, diagnoses, vaccines, and available treatments. Variants in the VOC category have, therefore, changed over time: Alpha, initially detected in the United Kingdom in December 2020; Beta, associated with South Africa since December 2020; Gamma, identified in Brazil in January 2021; Delta, originating in India and classified as of concern in May 2021. Currently, Omicron, known since November 2021, is the only circulating VOC, as defined by WHO [48].

Our results are in line with other published data suggesting that patients with the Delta variant, particularly if unvaccinated, might have increased ICU admission and need for invasive ventilatory support, more prolonged hospitalization, and higher mortality, as compared to the Alpha variant [49].

There were several limitations to our study. First, the relatively limited sample size constrains the generalizability of the results, particularly with regard to demographic factors. Second, data were collected retrospectively from electronic records, and there was no standard protocol for clinical registration or patient workup during ICU admission. Also, data on frailty, which is an important risk factor for ICU mortality, especially in the aged, were missing. One important additional limitation is that ICU admissions may not have occurred because of capacity strain and care limitations, such as high age and severe comorbidities of patients, which could skew the results. Finally, the absence of a control group does not allow the effect of comorbidities and differences in organ dysfunction to be assessed with accuracy and comprehensiveness.

## Conclusions

In this case series of critically ill patients with laboratory-confirmed SARS-CoV-2 infection, male sex and cardiovascular risk factors were common features. Approximately three-quarters of the patients required invasive mechanical ventilation and about 15% underwent tracheostomy for prolonged invasive mechanical ventilation and difficult weaning. Most patients received antibiotics for presumed bacterial respiratory co-infection, yet microbiological identification was achieved in a small proportion. Disorders of coagulation and fibrinolysis were frequent and severe, and about half of the patients received full-dose anticoagulant therapy. The overall 28-day mortality rate after ICU admission was high (40%).

We found significant differences in mortality based on age and sex. The patients that died within 28 days of ICU admission were more likely to have multiple comorbidities and cardiovascular risk factors. In addition, the proportions of invasively ventilated patients and patients treated with antimicrobials were significantly higher in the deceased group.

Early identification of risk factors for mortality is crucial in triage decision-making and for the proper utilization of resources. Moreover, physicians must manage unexpected complications as the disease progresses, particularly coagulation and fibrinolysis disorders. Studies are needed on the links between COVID-19 and age, cardiovascular risk factors, immunosuppression, and prognosis. Finally, improved methods for infection diagnosis, as well as effective antimicrobial stewardship programs, are required in this pandemic setting.
